# Biological Synthesis and Process Monitoring of an Aggregation-Induced Emission Luminogen-Based Fluorescent Polymer

**DOI:** 10.1021/jacsau.2c00436

**Published:** 2022-09-15

**Authors:** Chenchen Liu, Xuhui Bian, Ryan T. K. Kwok, Jacky W. Y. Lam, Lei Han, Ben Zhong Tang

**Affiliations:** † Department of Chemistry, Hong Kong Branch of Chinese National Engineering Research Center for Tissue Restoration and Reconstruction, Division of Life Science, and State Key Laboratory of Molecular Neuroscience, The Hong Kong University of Science and Technology, Clear Water Bay, Kowloon, Hong Kong 999077, China; ‡ College of Chemistry and Pharmaceutical Sciences, Qingdao Agricultural University, Qingdao, Shandong 266109, China; § Guangdong Provincial Key Laboratory of Luminescence from Molecular Aggregates, South China University of Technology, Guangzhou 510640, China; ∥ School of Science and Engineering, Shenzhen Key Laboratory of Functional Aggregate Materials, The Chinese University of Hong Kong, Shenzhen, Guangdong 518172, China

**Keywords:** fluorescent polymer, aggregation-induced emission, biological synthesis, process monitoring, bacterial cellulose

## Abstract

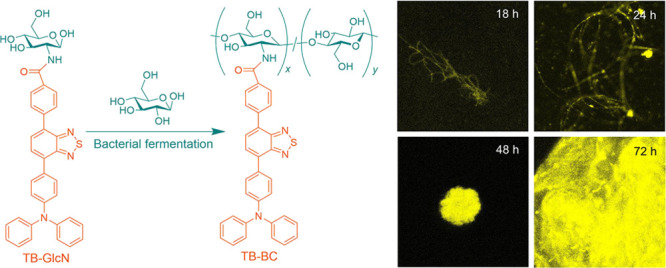

As the most abundant and renewable biopolymer on earth, cellulose can be functionalized for various advanced applications by chemical modification. In addition, fluorescent polymers with aggregation-induced emission (AIE) are generally prepared using chemical approaches, and the biosynthesis of AIE-active polymers are rarely investigated. Herein, fluorescent cellulose was successfully synthesized by bacterial fermentation, where glucosamine-modified AIE luminogen was incorporated into cellulose to achieve AIE-active biopolymers. Excitingly, real-time visualization of the synthetic process was realized, which is crucial for investigating the process of bacterial fermentation. The biosynthesized cellulose exhibited better performance with uniform fluorescence distribution and high stability, compared with that prepared by physical absorption. Additionally, fluorescent mats were fabricated by electrospinning of AIE-active cellulose, demonstrating its great potential applications in flexible display and tissue engineering.

## Introduction

Cellulose is the most abundant and renewable polysaccharide in nature and can be easily obtained from many plants including wood, grass, and cotton.^1−3^ Because of its advantages including low cost, excellent reproducibility, high flexibility, and good stretchability, cellulose has been historically used as a structural material in paper and textile industries for thousands of years.^4^ Recently, cellulose has also attracted much attention for its biomedical applications like drug delivery and tissue engineering, owing to its remarkable biocompatibility.^5−7^ Cellulose is significantly amphiphilic and insoluble in water and typical organic solvents because of the hydrophobic effects that keep cellulose crystals together,^8−10^ so the chemical modifications of cellulose are widely carried out in strong acidic or alkaline conditions on industrial scales. Recently, the dissolution behaviors of cellulose are also investigated in some unique solvent systems such as dimethyl sulfoxide/tetrabutylammonium fluoride, *N*,*N*-dimethylacetamide/LiCl, and ionic liquids.^11−14^ In nature, cellulose is synthesized by enzyme cellulose synthase in living organisms,^15^ which provides us a novel tool for the functionalization of cellulose through the biosynthesis process.

Nowadays, fluorescent materials have attracted increasing attention because of their promising applications in organic light-emitting diodes and biological fields.^16,17^ Cellulose is traditionally considered to be not fluorescent because of the lack of any chromophores. Although recent study demonstrates that it shows weak blue emission under UV irradiation because of the mechanism of clustering-triggered emission (CTE),^18^ the limited emission color and low quantum yield have restricted its applications. Therefore, the functionalization of cellulose with fluorescent dyes will expand its applications in many areas like flexible displays and tissue engineering. So far, some fluorescent celluloses have been synthesized by cotton or bacteria.^19,20^ However, the emission of traditional fluorescent dyes is largely decreased or even quenched in the solid state because of strong π–π interactions. Such an aggregation-caused quenching (ACQ) effect and small Stokes shifts have severely limited their applications as cellulose-based solid fluorescent materials.^21,22^ Therefore, the aim of this work is to develop the facile biosynthesis of fluorescent cellulose without the ACQ effect.

Fluorescent dyes showing aggregation-induced emission (AIE) are not emissive in solution but exhibit intense emission in the aggregate state because of the restriction of intramolecular motion.^23^ Thus, AIE luminogens (AIEgens) are promising dyes for functionalization of biopolymers and bioimaging.^24−26^ For example, fluorescent silks with full-color emissions could be prepared through bioconjugation with AIEgens and demonstrate great potential for long-term bioimaging.^27^ Nevertheless, the synthesis of AIE polymers through biosynthetic processes are rarely reported, so it is of great significance to monitor and investigate the in situ biosynthesis of AIE polymers.

**Scheme 1:**
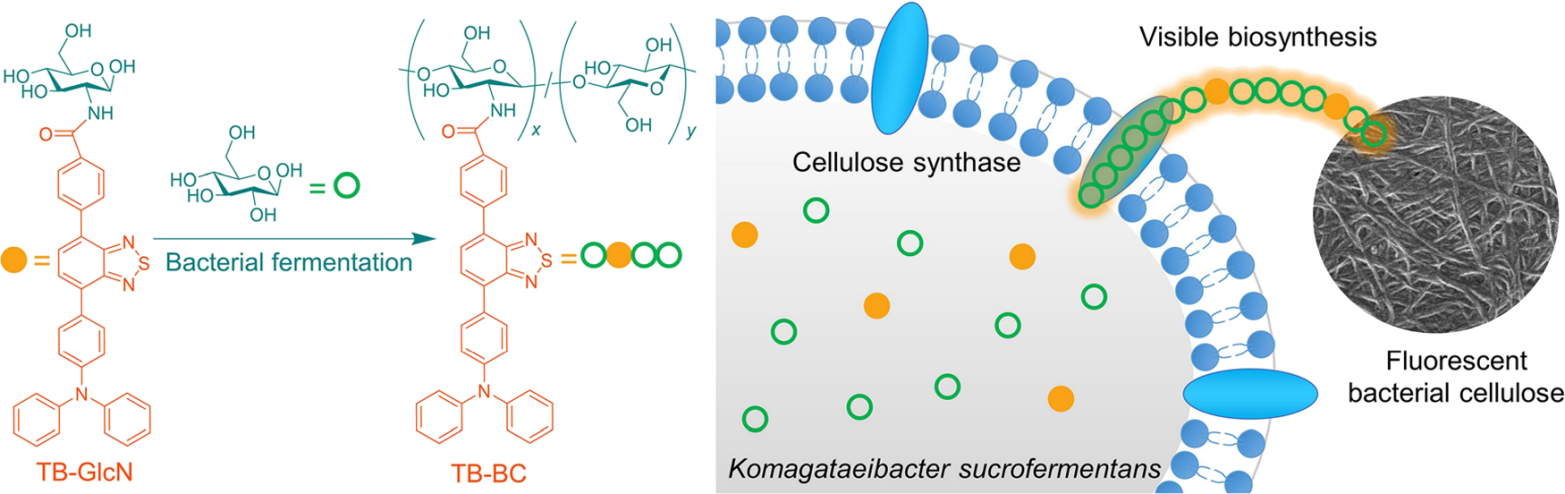
Schematic Illustration of the Biological Synthesis of TB–BC through Bacterial Fermentation

Herein, the biological synthesis of fluorescent cellulose with AIEgen was successfully realized through bacterial fermentation (Scheme 1). Normally, the morphology and growth of bacterial cellulose (BC) were investigated by scanning electron microscopy (SEM), but real-time monitoring of the whole biosynthetic process is hard to be realized.^28,29^ In this work, the AIEgen-modified glucosamine (named TB-GlcN) was incorporated into the main chain of cellulose, in order that in-situ and real-time visualization of the biosynthetic process was successfully performed by confocal laser scanning microscopy (CLSM). The biosynthesized fluorescent BC exhibited great superiority over that prepared by physical absorption in terms of brightness and stability. Additionally, fluorescent film based on AIEgen-modified cellulose and polyvinylpyrrolidone (PVP) was fabricated using the electrospinning method to demonstrate application potential in flexible display and tissue engineering. This work would not only open an avenue for biosynthesis of AIE polymers but also provide a real-time monitoring method for the biosynthetic process of cellulose.

## Results and Discussion

**Figure 1:**
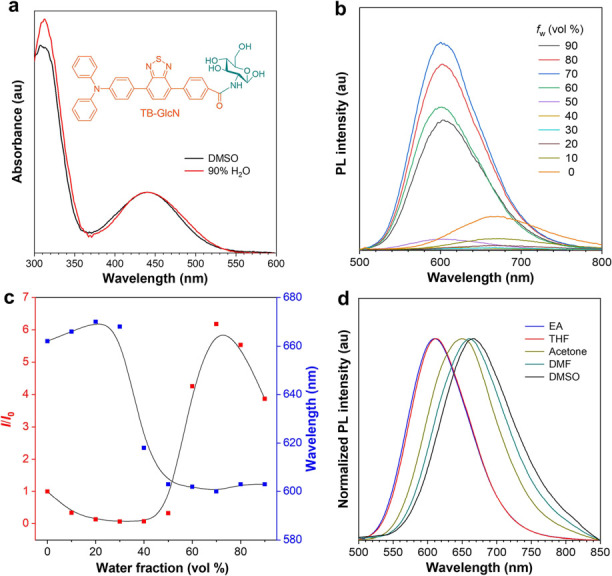
(a) Absorption spectra of TB-GlcN (10^–5^ M) in DMSO and DMSO/H_2_O (1/9, v/v) solutions. (b) PL spectra of TB-GlcN (10^–5^ M) in different DMSO/H_2_O mixtures with different water fractions (*f*_w_), λ_ex_ = 440 nm. (c) Plot of relative maximum emission intensity (*I/I*_0_) and maximum emission wavelength of TB-GlcN in different DMSO/H_2_O mixtures. (d) Normalized fluorescence spectra of TB-GlcN in varying solvents.

The AIEgen-functionalized glucosamine (TB-GlcN) was prepared according to the synthetic route shown in Scheme S1 in the Supporting Information. Triphenylamine and benzothiadiazole were selected as electron-donating and electron-withdrawing groups, respectively, to construct the yellow-emissive AIE unit. TB-GlcN was characterized using standard spectroscopic methods such as nuclear magnetic resonance (NMR) with satisfactory results (Figures S1–S11). Then, its photophysical properties were evaluated. As shown in Figure 1a, the UV spectrum of TB-GlcN measured in dimethyl sulfoxide (DMSO) solution exhibited a maximum at around 440 nm. In DMSO, TB-GlcN emitted red photoluminescence (PL) at 671 nm, which gradually red-shifted with decreased intensity upon addition of 30% water into the DMSO solution (Figure 1b, c) because of the twisted intramolecular charge transfer (TICT) effect.^30^ When the water fraction exceeded 50%, aggregates of TB-GlcN were formed and the PL peak blue shifted with increased intensity because of the restriction of molecular motion and suppression of the TICT effect. Evidently, TB-GlcN was AIE-active. In addition, emission peaks of TB-GlcN gradually red-shifted with the increase in solvent polarity (Figure 1d), which verified the TICT effect of TB-GlcN. The PL quantum yield of TB-GlcN powder was measured as 24.3%. Thus, TB-GlcN will be a promising fluorescent dye for biosynthesis of highly emissive fluorescent cellulose.

**Figure 2:**
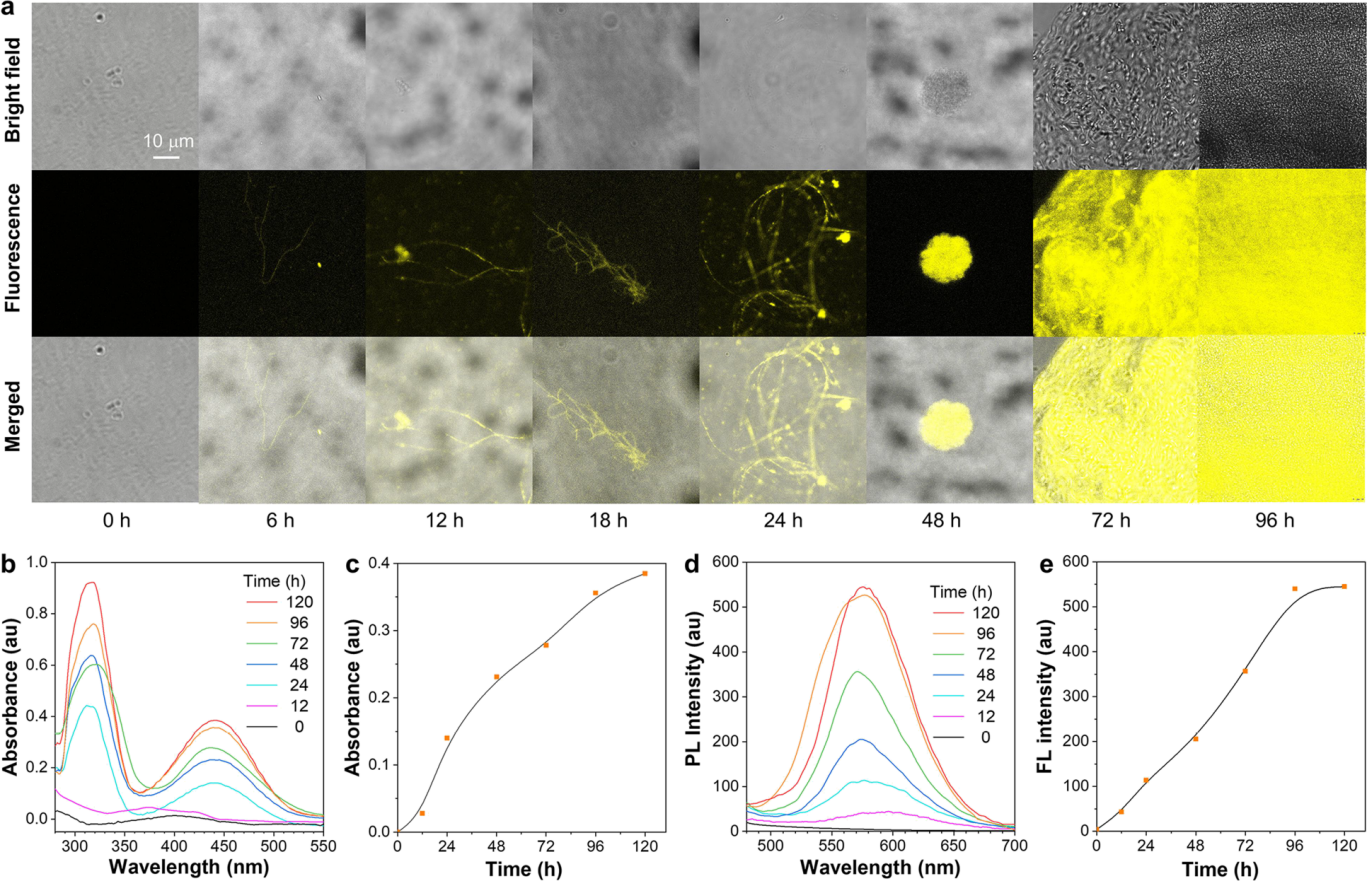
(a) CLSM images of TB–BC through bacterial fermentation under standard conditions (30 °C) for 0, 6, 12, 18, 24, 48, 72, and 96 h. Time-dependent (b) UV–vis absorption, (d) PL spectra, and (c, e) the plots of relative maximum intensities of TB–BC during bacterial fermentation. Scale bar corresponds to 10 μm.

Subsequently, TB-GlcN (1.5 × 10^–5^ M) and glucose were fed to the Hestrin–Schramm (H-S) culture medium where *Komagataeibacter sucrofermentans* (*K. sucrofermentans*) was employed for BC fermentation (Scheme 1).^31^ Yellow-emissive cellulose named TB–BC was obtained after 5-day incubation, which was treated with NaOH solution (2%, w/v) to remove the residual medium and microorganisms. The morphology of final BC fibers can be observed by SEM and atomic force microscopy (AFM), but in-situ visualization of the whole biosynthesis process is quite difficult because it is hard to track and separate the transparent cellulose fibers from the culture medium especially at the early stage. Benefiting from the high brightness and superior resolution of AIE-active TB-GlcN, real-time visualization of the biosynthesis process was realized by CLSM (Figure 2a). Although the ultrafine cellulose fibers were invisible in bright field within 24 h, we could still clearly visualize the biosynthesis process owing to the AIE property of TB-GlcN. Then, more cellulose fibers were gradually synthesized and entangled together to form cellulose nanoballs within 48 h, and uniform fluorescent cellulose film was finally formed and covered the whole field of view within 96 h. Additionally, time-dependent absorption and emission changes of TB–BC solutions were monitored during the incubation process. As shown in Figure 2b–e, the absorption and emission increased gradually with time and reached their maximum intensity at around 96 h, reflecting the rate of biological polymerization. Therefore, the introduction of the AIE monomer not only fabricated bright fluorescent cellulose materials but also provided a novel tool to visualize and investigate the biosynthesis process.

**Figure 3:**
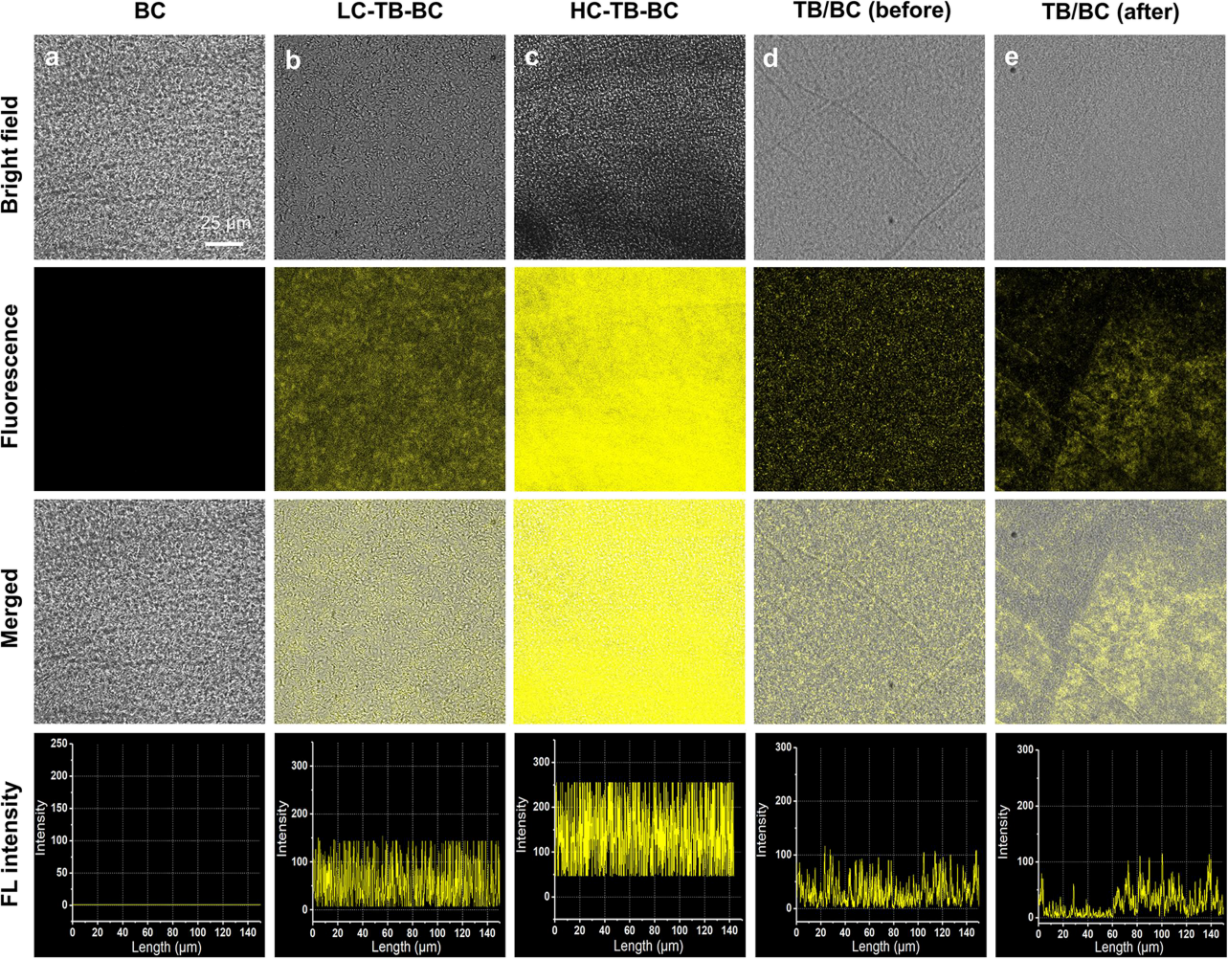
CLSM images and fluorescence intensities of (a) BC, (b) LC-TB–BC (low concentration of TB-GlcN, 1.5 **×** 10^–5^ M); (c) HC-TB–BC (high concentration of TB-GlcN, 3 **×** 10^–5^ M) after washing with NaOH (2%, w/v); and physically absorbed TB/BC (d) before and (e) after washing with NaOH (2%, w/v). The scale bar corresponds to 25 μm.

To research the effect of TB-GlcN concentration for biological synthesis of fluorescent cellulose, low concentration of TB-GlcN (LC-TB-GlcN, 1.5 × 10^–5^ M) and high concentration of TB-GlcN (HC-TB-GlcN, 3 × 10^–5^ M) were fed into the culture medium of *K. sucrofermentans* for bacterial fermentation, respectively. As shown in Figure 3a, no fluorescence was observed in BC by CLSM. The obtained LC-TB–BC and HC-TB–BC exhibited uniform yellow fluorescence distribution and HC-TB–BC exhibited higher fluorescence than LC-TB–BC (Figures 3b, c and S12), indicating that the fluorescence of TB–BC can be controlled by adjusting the concentration of the AIE monomer. As a contrast, physically absorbed TB/BC film was prepared by immersing the neat BC film into the H-S basic medium supplemented with TB-GlcN (1.5 × 10^–5^ M) for 5 days. Before washing with NaOH solution at 60 °C for 12 h, the emission of TB/BC film was quite low and the fluorescence distribution was not uniform (Figure 3d). Even worse, after washing with NaOH (2%, w/v), the physically absorbed TB-GlcN could be washed away from the BC film and fluorescence distribution of BC film was more unequal (Figures 3e and S13), suggesting low fluorescence stability.

**Figure 4:**
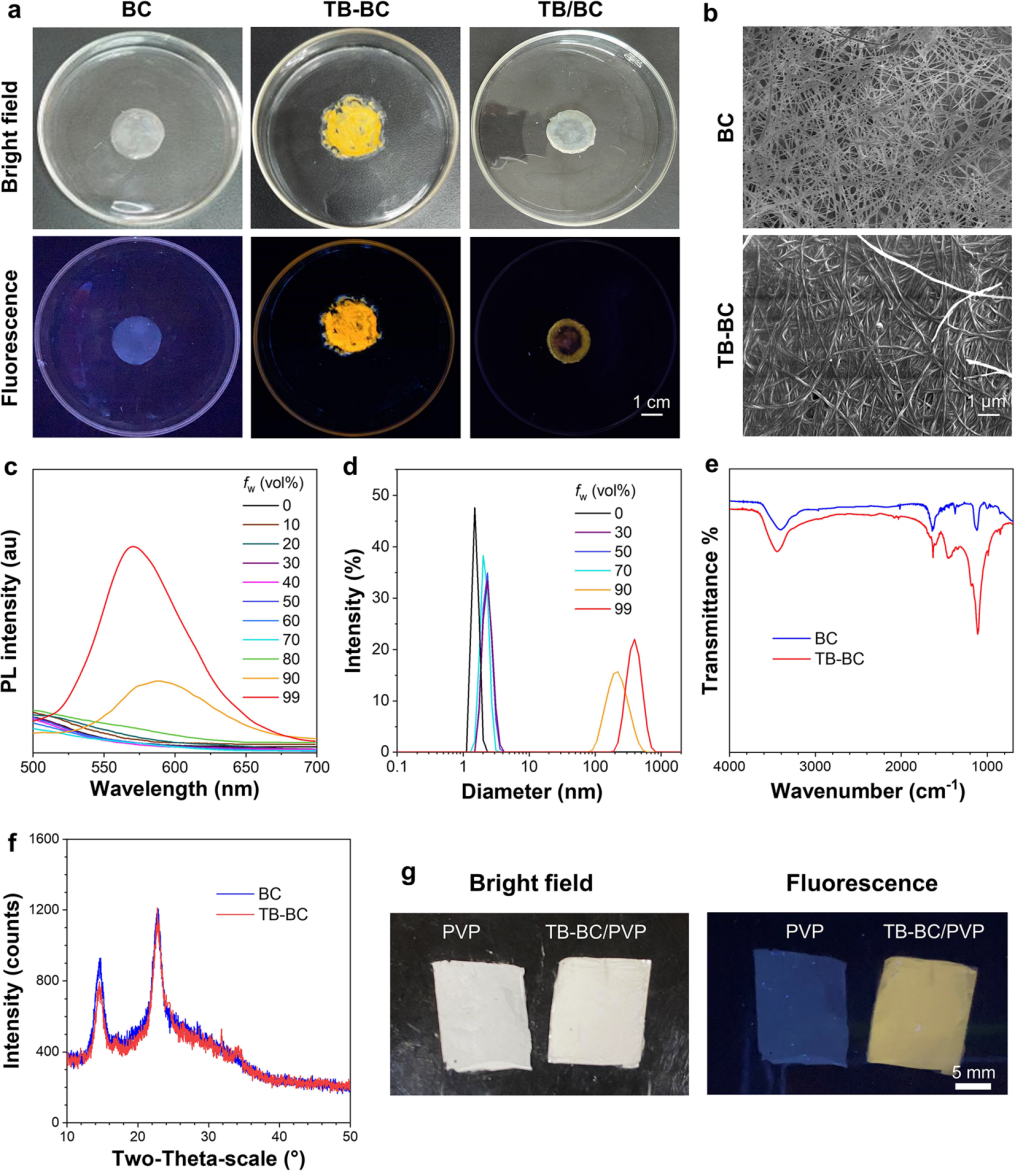
(a) Bright field and fluorescent photographs of BC, TB–BC, and TB/BC after wash, taken under 365 nm UV light illumination. (b) SEM images of BC and TB–BC. (c) PL spectra and (d) hydrodynamic size distribution of TB–BC (0.1 mg/mL) in THF/H_2_O mixtures with different water fractions (*f*_w_), λ_ex_ = 430 nm. (e) FT-IR, ATR, and (f) XRD spectra of BC (blue) and TB–BC (red). (g) Bright field and fluorescent photographs of PVP and TB–BC/PVP mats prepared by electrospinning.

Furthermore, BC, TB–BC, and TB/BC films after wash were observed under bright field and UV light (Figures 4a and S14). The neat BC showed weak blue fluorescence with the quantum yield of 4.7% probably due to the CTE mechanism. In contrast, bright yellow emission was observed in TB–BC with a fluorescence quantum yield of 34.6%. Expectedly, the TB/BC after wash film exhibited weak and nonuniform yellow fluorescence, and the inner part was almost dark under UV light because it was difficult for TB-GlcN to penetrate into the BC film. In addition, the SEM images demonstrated the network structures of BC and TB–BC films, and no significant difference in the diameter of BC and TB–BC fibers was observed (Figures 4b and S15). The PL spectra (Figure 4c) and hydrodynamic distribution (Figure 4d) measurements evaluated that the fluorescence of TB–BC dramatically increased after the formation of aggregates in aqueous solution, which confirmed the AIE property of the TB–BC polymer. The Fourier transform infrared (FT-IR) spectra of BC and TB–BC were also measured (Figure 4e), and both exhibited typical absorption peaks of cellulose at 3441 cm^–1^ (OH stretching), 1634 cm^–1^ (C=O stretching), and 1108 cm^–1^ (antisymmetric stretching of C–O–C).^32,33^ In addition, an obvious peak appeared at 1452 cm^–1^ (C–N stretching in CO–NH) in TB–BC, indicating that the TB-GlcN molecule was successfully incorporated to the cellulose. The X-ray diffraction (XRD) spectra of BC and TB–BC (Figure 4f) showed similar absorption peaks at 14.8° and 22.9°, which reflected to the (110) and (200) planes of the I-β crystal of cellulose, respectively,^34^ indicating that the incorporation of TB-GlcN had no obvious influence on the crystal structure of BC.

Recently, electrospun nanofibers based on cellulose have attracted much attention for biomedical applications such as tissue engineering scaffolds, wound healing, and drug carriers, owing to their high specific surface area, interconnected porosity, and excellent biocompatibility.^35−38^ Moreover, BC has higher purity and biocompatibility than plant-produced cellulose that contains hemicellulose, lignin, and pectin.^39^ As a biocompatible polymer, polyvinylpyrrolidone (PVP) has been widely utilized for medical and pharmaceutical applications.^40^ For the proof of concept, TB–BC was employed for the fabrication of fluorescent mats by electrospinning technology with PVP that is extensively employed as a polymer carrier to improve the film formation ability and the mechanical property.^41,42^ As shown in Figures 4g and S16, the PVP nanofiber mat showed weak blue fluorescence probably due to CTE,^43^ while bright yellow emission of the flexible TB–BC/PVP nanofiber mat could be observed under UV irradiation. By virtue of its excellent flexibility and biocompatibility, the fluorescent TB–BC/PVP nanofiber mat would be a promising candidate as a visible scaffold for tissue engineering.

## Conclusions

In summary, the biological synthesis of fluorescent cellulose was realized by using glucosamine-modified AIEgen (TB-GlcN) and glucose as the substrate under bacterial fermentation, and the biosynthesis process could be clearly visualized by confocal imaging. The as-synthesized fluorescent cellulose (TB–BC) exhibited uniform fluorescence distribution, and the emission intensity could be facilely controlled by adjusting the concentration of TB-GlcN. In contrast, TB/BC fabricated by physical absorption showed poor stability and nonuniform color distribution. For the proof of concept, the TB–BC/PVP mat was successfully fabricated using the electrospinning technique, demonstrating great potential in tissue engineering materials with excellent flexibility and biocompatibility. This work would provide a novel tool to biosynthesize AIE polymers and visualize the biosynthesis process of cellulose.

## Methods

### Microbial Fermentation and Preparation of BC

*K. sucrofermentans* was inoculated in 7% (v/v) H–S basic medium and cultured at 30 °C for 5 days. The obtained BC samples were treated with NaOH solution (2%, w/v) for 12 h at 60 °C to remove bacterial debris and then washed thoroughly with ultrapure water until neutral pH. All samples were dried at room temperature in vacuum.

### Preparation of TB–BC by Bacterial Fermentation

*K. sucrofermentans* was cultured with H–S basic medium supplemented with TB-GlcN (low concentration, 1.5 × 10^–5^ M or high concentration, 3 × 10^–5^ M) at 30 °C for 5 days. Then, TB–BC was treated with NaOH solution (2%, w/v) at 60 °C for 12 h and then washed with ultrapure water thoroughly until no fluorescence was detected in the residual water. All samples were dried at room temperature in vacuum.

### Preparation of TB/BC before and after Wash Films

TB/BC before wash film was obtained by immersing neat BC film into the H–S basic medium supplemented with TB-GlcN (1.5 × 10^–5^ M) for physical adsorption at 30 °C for 5 days. Then, TB/BC after wash film was obtained by treating TB/BC before wash film with NaOH solution (2%, w/v) at 60 °C for 12 h and washed with deionized water until no obvious fluorescence was detected in the residual water. All samples were dried at room temperature in vacuum.

### Preparation of TB–BC/PVP Mats by Electrospinning

First, polyvinylpyrrolidone (PVP) (2 g) was dissolved in ethanol (4 mL) and stirred for 30 min. Then, LC-TB–BC solution (3 mg in 1 mL THF) was added into above PVP solution under continuous stirring. The electrospinning experiment was carried out on an electrostatic spinning instrument (DT-200, Dalian Dingtong Technology Development Co., Ltd.). In a typical procedure, the electrospinning solution was put into a syringe and then pumped into the spray nozzle with a propulsion speed of 0.005 mm s^–1^. The positive voltage (10 kV) was applied to the polymer solution via a stainless-steel syringe needle. The distance between the tip of the needle and the collector was maintained at 152 cm. The electrospun polymer fibers were collected on an aluminum foil.

## Supporting Information Available

General information about materials; synthetic procedures; ^1^H NMR; ^13^C NMR and high-resolution mass spectra of the compounds; SEM photographs of fluorescent BC samples; and fluorescence photographs of TB–BC/PVP mats through electrospinning (PDF)
