# Supplementary material for: Biological Synthesis and Process Monitoring of an Aggregation-Induced Emission Luminogen-Based Fluorescent Polymer
Source: JACS Au. 2022 Sep 15;2(9):2162–8. doi: 10.1021/jacsau.2c00436 (PMC9516714; doi:10.1021/jacsau.2c00436)
Supplement: Supplementary file 1 — au2c00436_si_001.pdf [file au2c00436_si_001.pdf]

Supporting Information

Biological Synthesis and Process Monitoring of an Aggregation-Induced Emission Luminogen-Based Fluorescent Polymer

Chenchen Liu[†,‡], Xuhui Bian[†,§], Ryan T. K. Kwok[‡], Jacky W. Y. Lam[‡], Lei Han[*,§,∥] and Ben Zhong Tang*[,‡,⊥]

[‡]Department of Chemistry, Hong Kong Branch of Chinese National Engineering Research Center for Tissue Restoration and Reconstruction, Division of Life Science, and State Key Laboratory of Molecular Neuroscience, The Hong Kong University of Science and Technology, Clear Water Bay, Kowloon, Hong Kong, China
[§]College of Chemistry and Pharmaceutical Sciences, Qingdao Agricultural University, Qingdao, Shandong 266109, China
[∥]Guangdong Provincial Key Laboratory of Luminescence from Molecular Aggregates, South China University of Technology, Guangzhou 510640, China
[⊥]School of Science and Engineering, Shenzhen Key Laboratory of Functional Aggregate Materials, The Chinese University of Hong Kong, Shenzhen, Guangdong 518172

[†] C. Liu and X. Bian contributed equally to this work.
*Correspondence should be addressed to Prof. L. Han (hanlei@qau.edu.cn) and Prof. B. Z. Tang (tangbenz@cuhk.edu.cn).

Table of Contents

General information S3
Materials S3
Apparatus S3
Strains and culture medium S3
Synthesis S4-S11
Scheme S1. Synthetic route to TB-GlcN S4-S5
Figure S1-S11. ^{1}H NMR, ^{13}C NMR and HRMS Spectra.................................... S6-S11
Scheme S2. Biological synthesis of TB–BC through bacterial fermentation S11
Figure S12. Absorption and PL spectra of HC-TB–BC, LC-TB–BC and BC S12
Figure S13. Absorption and PL spectra of TB/BC before and after wash films........S12
Figure S14. Normalized PL spectra of TB-GlcN powder, BC and TB–BC films S12
Figure S15. SEM photographs of BC, HC-TB–BC and LC-TB–BC.........................S13
Figure S16. The fluorescence photo of TB–BC/PVP mat through electrospinning ..S13
Reference S14

General information

1. Materials

Yeast extract and tryptone were purchased from Oxoid (USA). Na_2HPO_4 was purchased from Shanghai Titan Scientific Co., Ltd. (China). Sodium hydroxide (NaOH), citric acid monohydrate, and glucose were purchased from Sinopharm Chemical Reagent Co., Ltd. (China). All the other chemicals were purchased from Sigma-Aldrich (USA) or Sinopharm Chemical Reagent Co., Ltd. (China), and used directly without further purification. Ultrapure water (18.0 MΩ cm) was prepared by a Milli-Q system (Millipore, Germany) and used throughout.

2. Apparatus

^{1}H NMR and ^{13}C NMR spectra were measured by the Bruker ARX 400 NMR spectrometer. High resolution mass spectra (HRMS) were measured by GCT premier CAB048 mass spectrometer operating in a MALDI–TOF mode. UV–Vis absorption spectra were measured on PerkinElmer Lambda 365 Spectrophotometer. Photoluminescence (PL) spectra were measured by Edinburgh FLS980 Spectrofluorometer. Absolute fluorescence quantum yields were measured by Hamamatsu quantum yield spectrometer C11347 Quantaurus QY. Confocal laser scanning microscopy (CLSM) TCSsp5II (Agilent, USA) was conducted to monitor the production process of TB–BC and observe different BC fibers, the fluorescent images were taken at λ_{ex} = 458 nm and λ_{em} = 500–650 nm. To research the morphologies of various BC samples, samples were coated by platinum sputter, and then observed on Scanning electron microscope (SEM) S-4800 (Hitachi, Japan). After drying in vacuum, Fourier-transform infrared (FTIR) spectra of BC samples were recorded on a FTIR Spectrometer Nicolet iS10 (Thermo Fisher, USA) at 25 °C and 30% relative humidity. All spectra were recorded in the spectral range between 4000 and 700 cm^{-1} with a resolution of 0.5 cm^{-1}. The X-ray diffraction (XRD) spectra of processed dried samples were analyzed on a diffractometer D8 Advance (Bruker, Germany) using Cu Kα radiation (λ = 0.154 nm) at 60 kV and 60 mA.

3. Strains and culture medium

The bacterial strain *Komagataeibacter sucrofermentans* (*K. sucrofermentans*) was used for BC fermentation. Hestrin–Schramm (H-S) medium containing glucose (25 g L^{-1}), yeast extract (5 g L^{-1}), peptone (5 g L^{-1}), citric acid monohydrate (1.2 g L^{-1}) and Na_2HPO_4 (2.7 g L^{-1}) were sterilized at 115 °C for 30 min and cooled to room temperature before inoculation with the bacteria.

Synthesis

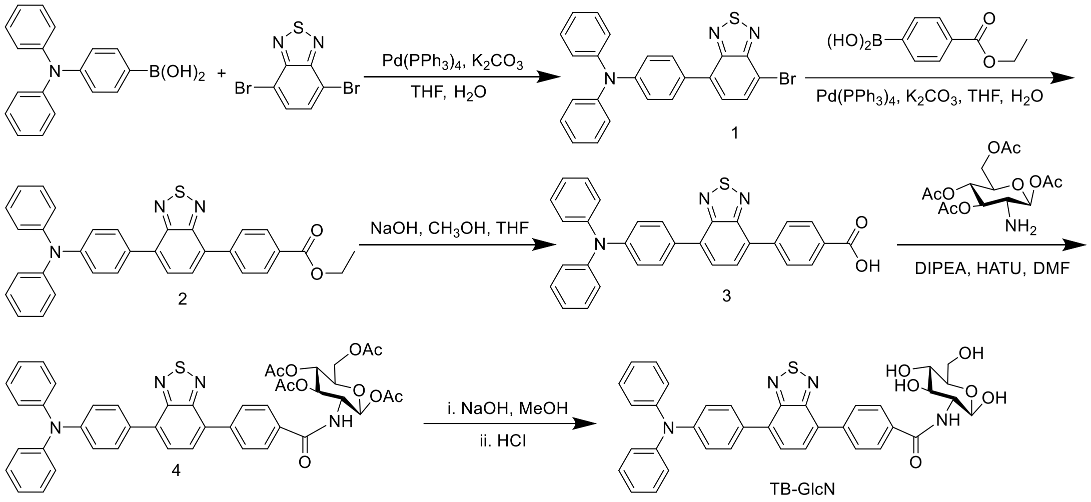

Scheme S1. Synthetic route to TB-GlcN.

The compound 1 was synthesized according to the previous literature.[1]

Synthesis of ethyl 4-(7-(4-(diphenylamino)phenyl)benzo[c][1,2,5]thiadiazol-4-yl) benzoate (compound 2). In a 100 mL two-neck round-bottom flask, 4-(7-bromobenzo [c][1,2,5]thiadiazol-4-yl)-N,N-diphenylaniline (compound 1) (2.29 g, 5 mmol), (4-(ethoxy carbonyl)phenyl)boronic acid (970 mg, 5 mmol) and $Pd(PPh_3)_4$ (30 mg, 0.026 mmol) were dissolved in THF (60 mL) and K_2CO_3 solution (2 M, 5 mL) under the protection of N_2, then the mixture was heated to 80 °C and stirred for 24 h. After that, the reaction mixture was cooled to room temperature and exacted with DCM (50 mL × 3), then the organic layer was dried over $MgSO_4$ and concentrated. The crude product was purified by silica gel chromatography with hexane/ethyl acetate (3:1, v/v) to obtain compound 2 as yellow power (2.21 g, 84%). ^{1}H NMR (400 MHz, Chloroform-*d*) δ 8.24 – 8.18 (m, 2H), 8.08 – 8.03 (m, 2H), 7.91 – 7.86 (m, 2H), 7.84 – 7.75 (m, 2H), 7.30 (dd, *J* = 8.5, 7.2 Hz, 4H), 7.24 – 7.17 (m, 6H), 7.11 – 7.04 (m, 2H), 4.43 (q, *J* = 7.1 Hz, 2H), 1.43 (t, *J* = 7.1 Hz, 3H). ^{13}C NMR (101 MHz, Chloroform-*d*) δ 166.46, 154.07, 154.00, 148.32, 147.43, 141.83, 133.76, 131.40, 130.55, 130.03, 129.84, 129.42, 129.12, 128.79, 127.10, 125.03, 123.47, 122.73, 61.07, 14.40. HRMS (MALDI-TOF, m/z): [M] calcd for $C_{33}H_{25}N_3O_2S$ 527.1667, found 527.1671.

Synthesis of 4-(7-(4-(diphenylamino)phenyl)benzo[c][1,2,5]thiadiazol-4-yl) benzoic acid (compound 3). In a 100 mL two-neck round-bottom flask, ethyl 4-(7-(4-(diphenylamino) phenyl)benzo[c][1,2,5]thiadiazol-4-yl)benzoate (compound 2) (1.05 g, 2 mmol) and NaOH (0.4 g, 10 mmol) were added in CH_3OH (25 mL) and THF (25 mL) solution, then the mixture was heated to 80 °C and stirred for 12 h. After that, the reaction mixture was cooled to room temperature and exacted with DCM (50 mL × 3), then the organic layer was dried over $MgSO_4$ and concentrated. The crude product was purified by silica gel chromatography with hexane/ethyl acetate (1:4, v/v) to obtain compound 3 as yellow power (878.5 mg, 88%). ^{1}H NMR (400 MHz, Chloroform-*d*) *δ* 8.30 (d, *J* = 8.1 Hz, 2H), 8.13 (d, *J* = 8.1 Hz, 2H), 7.92 (d, *J* = 8.3 Hz, 2H), 7.88 (d, *J* = 7.4 Hz, 1H), 7.82 (d, *J* = 7.3 Hz, 1H), 7.33 (t, *J* = 7.7 Hz,

4H), 7.24 (t, *J* = 8.5 Hz, 6H), 7.11 (t, *J* = 7.3 Hz, 2H). HRMS (MALDI-TOF, m/z): [M] calcd for $C_{31}H_{21}N_3O_2S$ 499.1354, found 499.1355.

Synthesis of (2*R*,3*S*,5*R*,6*S*)-6-(acetoxymethyl)-3-(4-(7-(4-(diphenylamino)phenyl) benzo[c][1,2,5]thiadiazol-4-yl)benzamido)tetrahydro-2H-pyran-2,4,5-triyltri acetate (compound 4). In a 50 mL two-neck round-bottom flask, 4-(7-(4-(diphenylamino) phenyl)benzo[c][1,2,5]thiadiazol-4-yl)benzoic acid (compound 3) (499 mg, 1 mmol), (2*S*,3*R*,4*S*,5*S*,6*R*)-6-(acetoxymethyl)-3-aminotetrahydro-2H-pyran -2,4,5-triyl triacetate (416 mg, 1.2 mmol), N,N-Diisopropylethylamine (1mL) and 2-(7-Azabenzotriazol-1-yl)-N,N,N',N'-tetramethyluronium hexafluorophosphate (456 mg, 1.2 mmol) were dissolved in DMF (50 mL) under the protection of N_2, then the mixture was heated to 120 °C and stirred for 24 h. After that, the reaction mixture was cooled to room temperature and exacted with DCM (50 mL × 3), then the organic layer was dried over $MgSO_4$ and concentrated. The crude product was purified by silica gel chromatography with hexane/ethyl acetate (1:1, v/v) to obtain compound 4 as yellow power (604.4 mg, 73%). ^{1}H NMR (400 MHz, Chloroform-*d*) δ 7.98 (d, *J* = 8.1 Hz, 2H), 7.86 (dd, *J* = 11.9, 8.3 Hz, 4H), 7.70 (s, 2H), 7.30 (t, *J* = 7.7 Hz, 5H), 7.20 (t, *J* = 6.3 Hz, 6H), 7.08 (t, *J* = 7.3 Hz, 2H), 6.62 (d, *J* = 9.5 Hz, 1H), 5.86 (d, *J* = 8.8 Hz, 1H), 5.40 (t, *J* = 10.1 Hz, 1H), 5.26 (t, *J* = 9.7 Hz, 1H), 4.66 (q, *J* = 9.6 Hz, 1H), 4.33 (dd, *J* = 12.5, 4.7 Hz, 1H), 4.19 (dd, *J* = 12.5, 2.3 Hz, 1H), 3.93 (ddd, *J* = 10.0, 4.9, 2.2 Hz, 1H), 2.14 – 2.08 (m, 9H), 2.06 (s, 3H). ^{13}C NMR (101 MHz, Chloroform-*d*) δ 171.74, 170.73, 169.32, 166.95, 148.35, 147.39, 133.76, 132.89, 130.95, 130.00, 129.48, 129.42, 128.62, 127.28, 126.98, 125.05, 123.49, 122.66, 92.86, 73.20, 72.86, 67.86, 61.82, 53.33, 20.92, 20.77, 20.73, 20.61. HRMS (MALDI-TOF, m/z): [M] calcd for $C_{45}H_{40}N_4O_{10}S$ 828.2465, found 828.2461.

Synthesis of 4-(7-(4-(diphenylamino)phenyl)benzo[c][1,2,5]thiadiazol-4-yl)-N-((2*S*,3*S*,4*R*,5*R*,6*S*)-2,4,5-trihydroxy-6-(hydroxymethyl)tetrahydro-2H-pyran-3-yl) benzamide (TB-GlcN). In a 50 mL two-neck round-bottom flask, (2*R*,3*S*,5*R*,6*S*)-6-(acetoxymethyl)-3-(4-(7-(4-(diphenylamino)phenyl)benzo[c][1,2,5]thiadiazol-4-yl) benzamido)tetrahydro-2H-pyran-2,4,5-triyl triacetate (compound 4) (165.64 mg, 0.2 mmol) and NaOH (80 mg, 2 mmol) were added in MeOH (30 mL) and stirred at room temperature for 2h, then pH was adjusted to 7 with HCl solution (0.01M). After that, the reaction mixture was exacted with DCM (50 mL × 3) and the organic layer was dried over $MgSO_4$ and concentrated. The crude product was purified by silica gel chromatography with DCM/MeOH (5:1, v/v) to obtain compound TB-GlcN as yellow power (69.9 mg, 53%). ^{1}H NMR (400 MHz, DMSO-d_6) δ 8.18 – 8.07 (m, 4H), 8.06 – 7.95 (m, 4H), 7.37 (dd, *J* = 8.7, 7.1 Hz, 4H), 7.13 (dd, *J* = 7.8, 2.3 Hz, 8H), 6.55 (dd, *J* = 41.2, 5.4 Hz, 1H), 5.13 (t, *J* = 3.9 Hz, 1H), 4.98 (dd, *J* = 10.6, 5.4 Hz, 1H), 4.71 (dd, *J* = 35.7, 6.3 Hz, 1H), 4.52 (dt, *J* = 37.2, 5.8 Hz, 1H), 4.11 (q, *J* = 5.2 Hz, 1H), 3.90 – 3.70 (m, 2H), 3.68 – 3.65 (m, 1H), 3.53 (dt, *J* = 11.9, 6.0 Hz, 1H), 3.22 (d, *J* = 5.3 Hz, 1H), 3.17 (d, *J* = 5.2 Hz, 1H). ^{13}C NMR (101 MHz, DMSO-d_6) δ 166.58, 153.83, 148.09, 147.35, 139.86, 132.82, 131.12, 130.76, 130.73, 130.19, 129.38, 129.22, 128.21, 128.06, 127.81, 125.04, 124.11, 122.73, 90.94, 72.65, 71.54, 70.58, 61.64, 55.94. HRMS (MALDI-TOF, m/z): [M] calcd for $C_{37}H_{32}N_4O_6S$ 660.2043, found 660.2054.

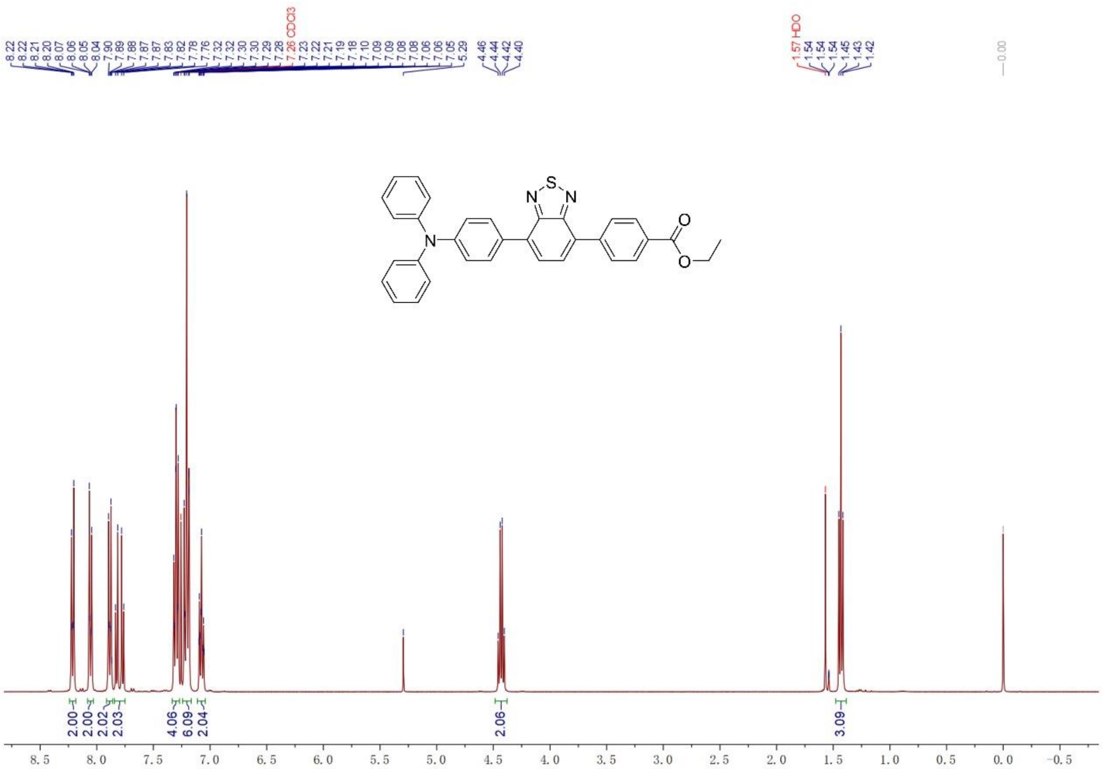

Figure S1. ^{1}H NMR spectrum of compound 2 in $CDCl_3$.

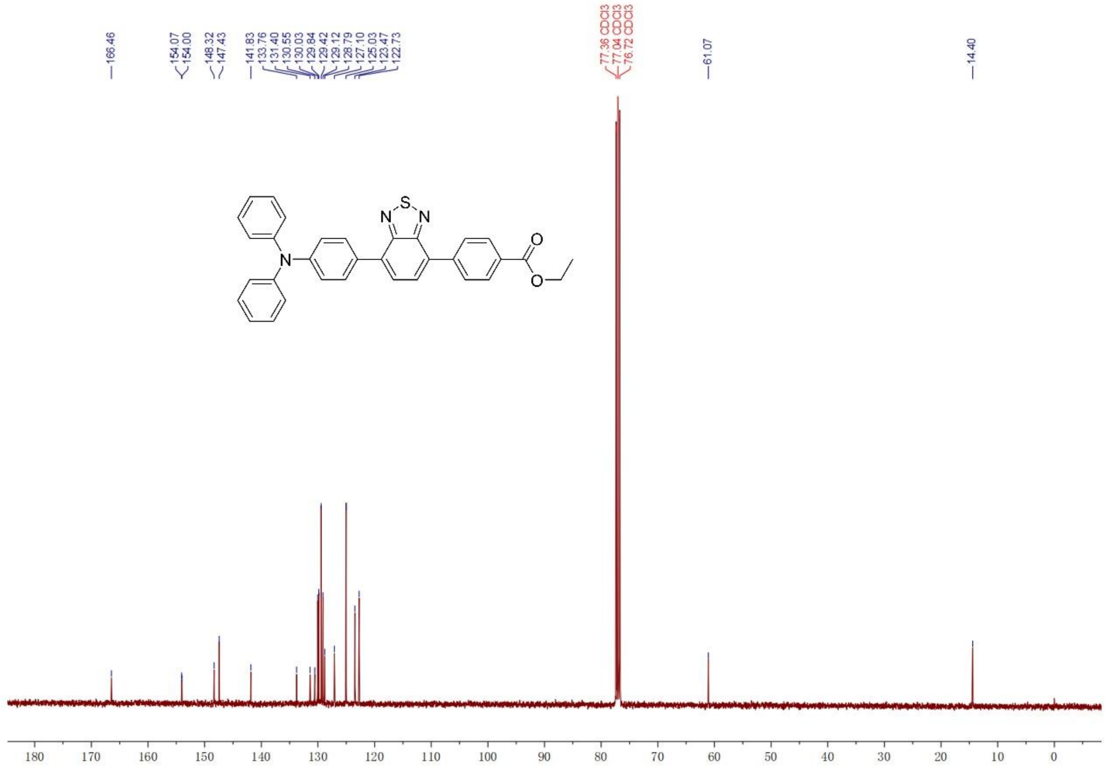

Figure S2. ^{13}C NMR spectrum of compound 2 in $CDCl_3$.

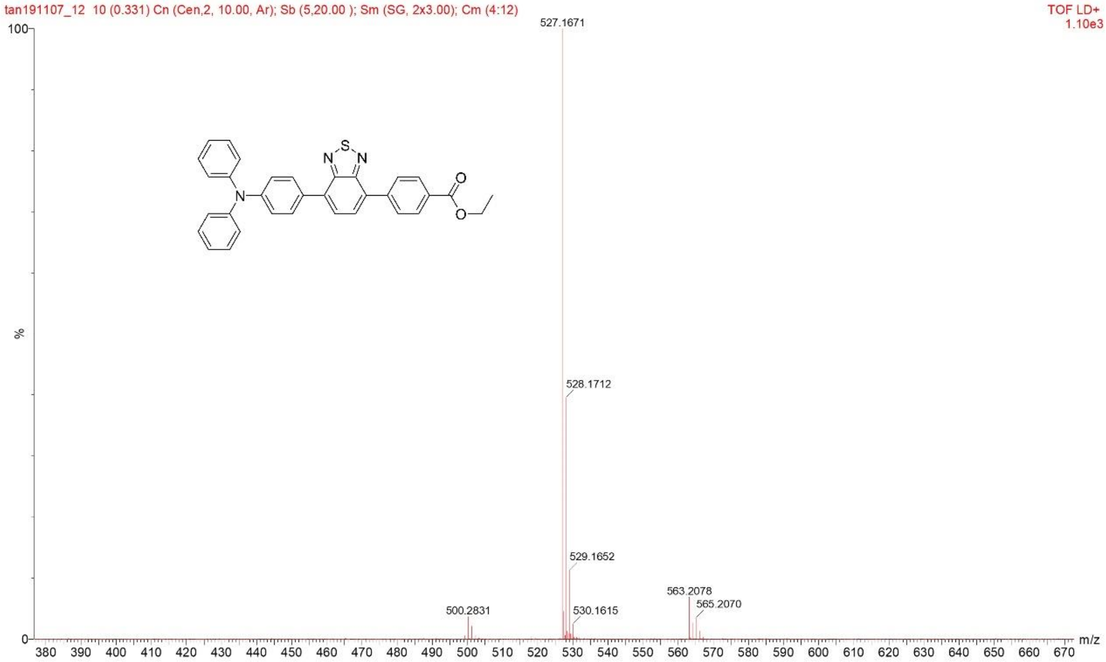

Figure S3. HRMS of compound 2.

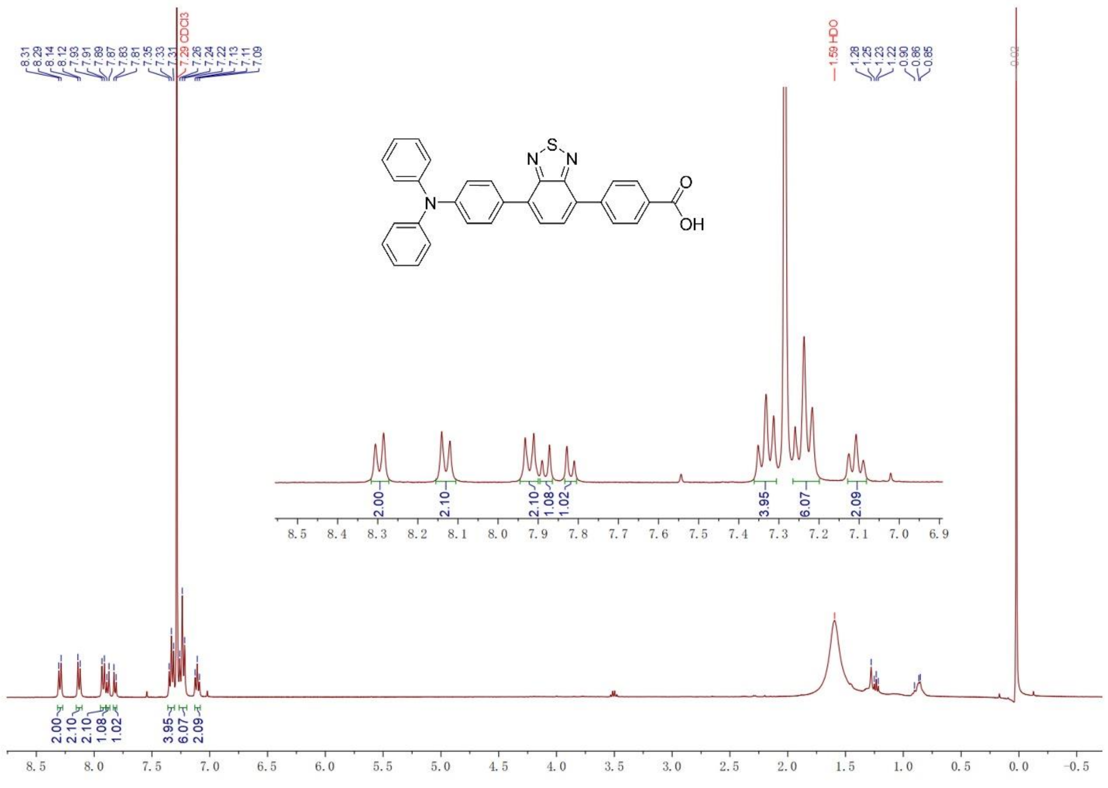

Figure S4. ^{1}H NMR spectrum of compound 3 in $CDCl_3$.

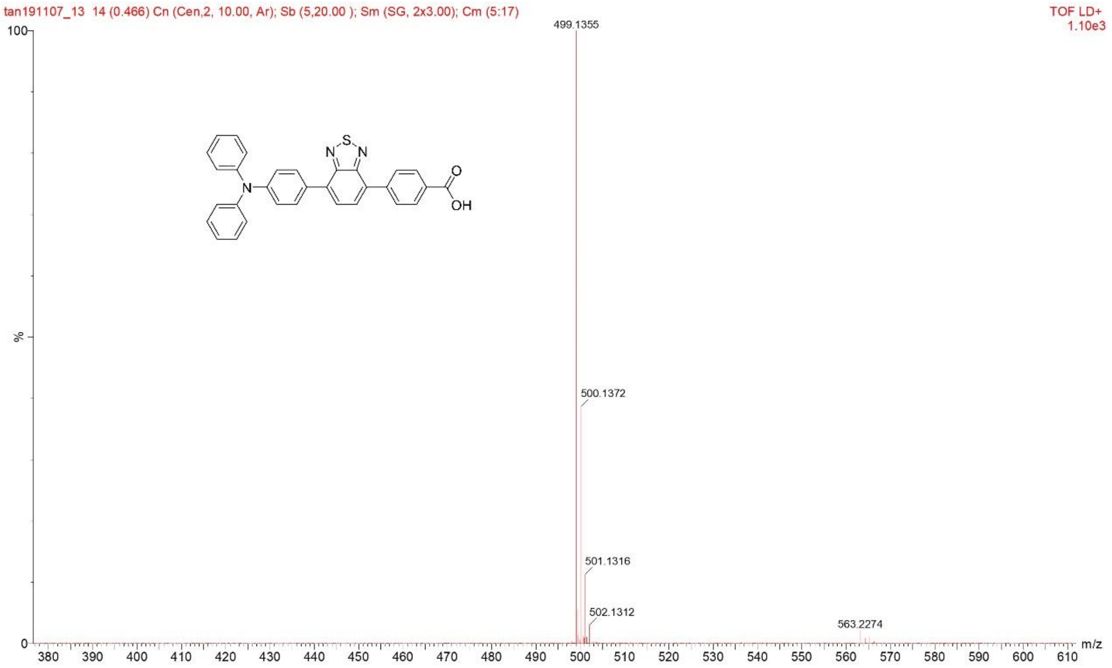

Figure S5. HRMS of compound 3.

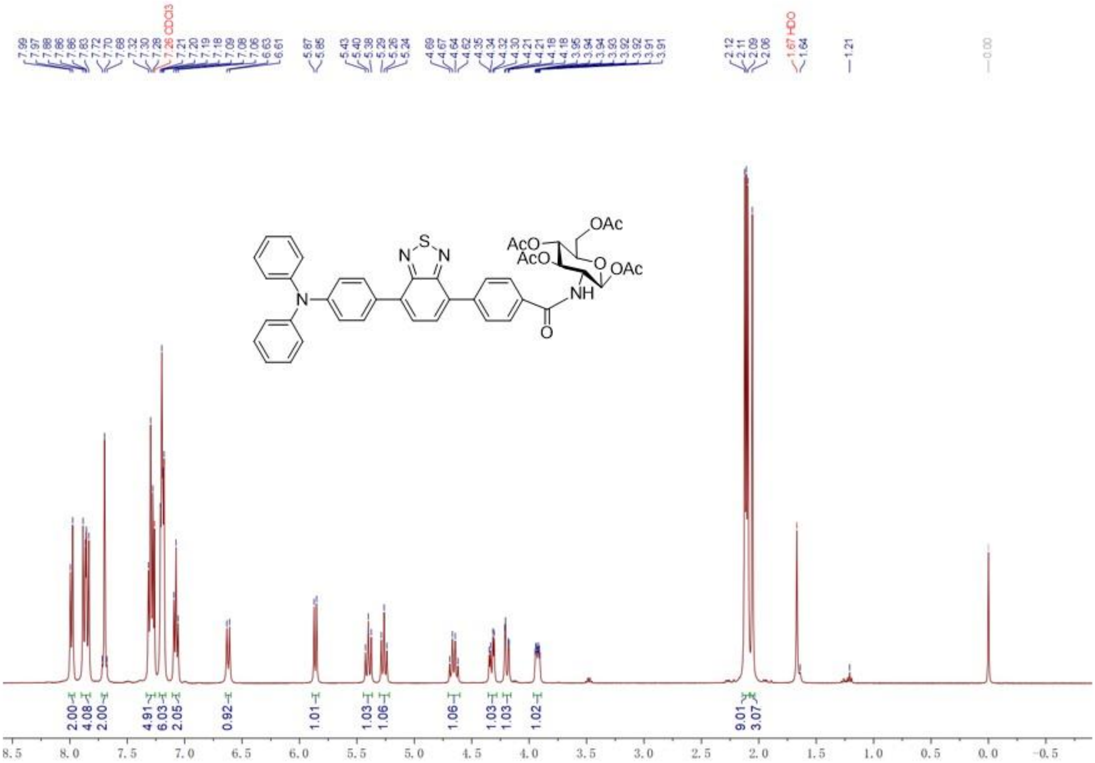

Figure S6. ^{1}H NMR spectrum of compound 4 in $CDCl_3$.

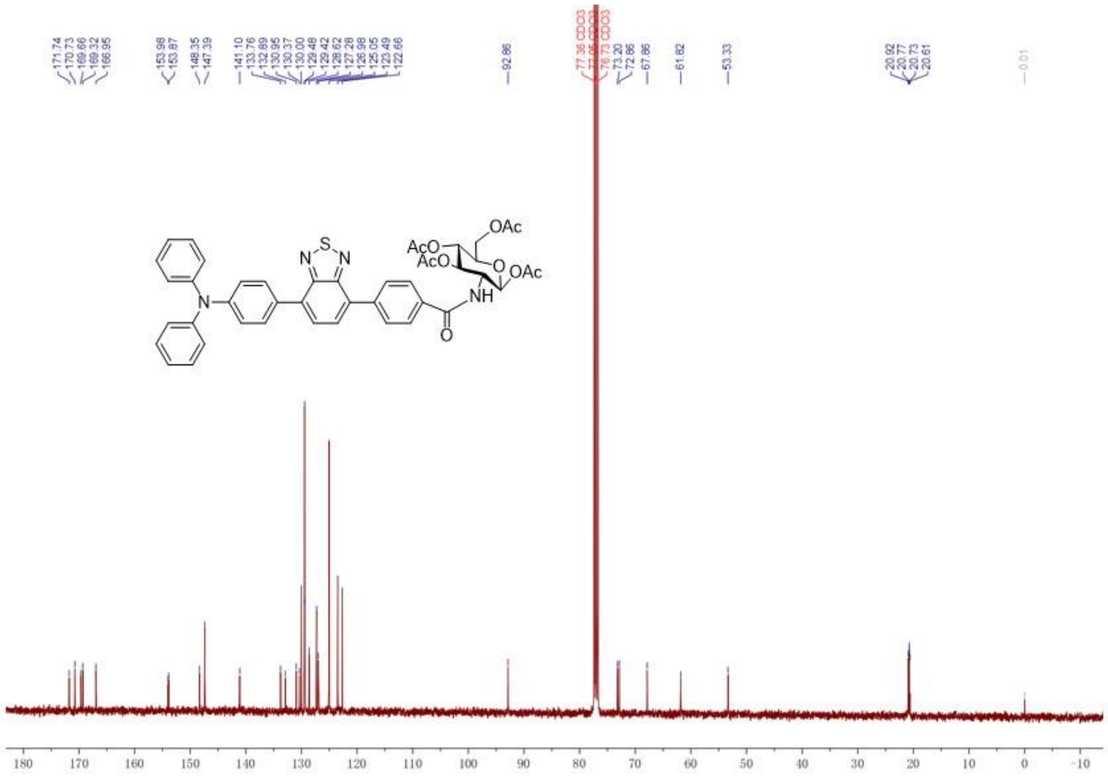

Figure S7. ^{13}C NMR spectrum of compound 4 in $CDCl_3$.

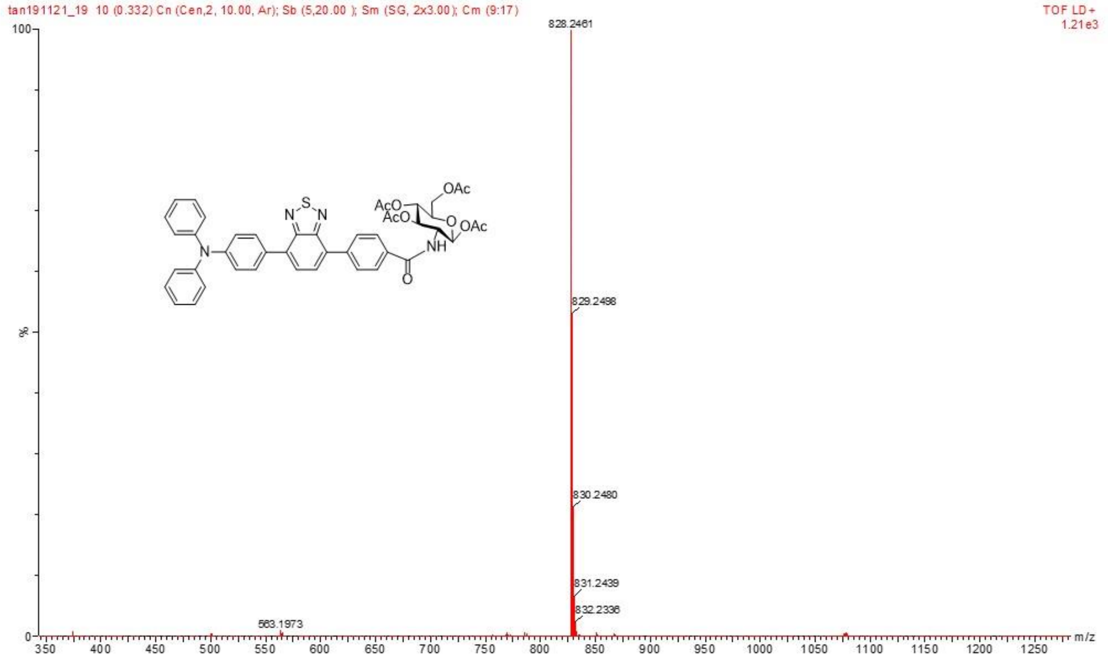

Figure S8. HRMS of compound 4.

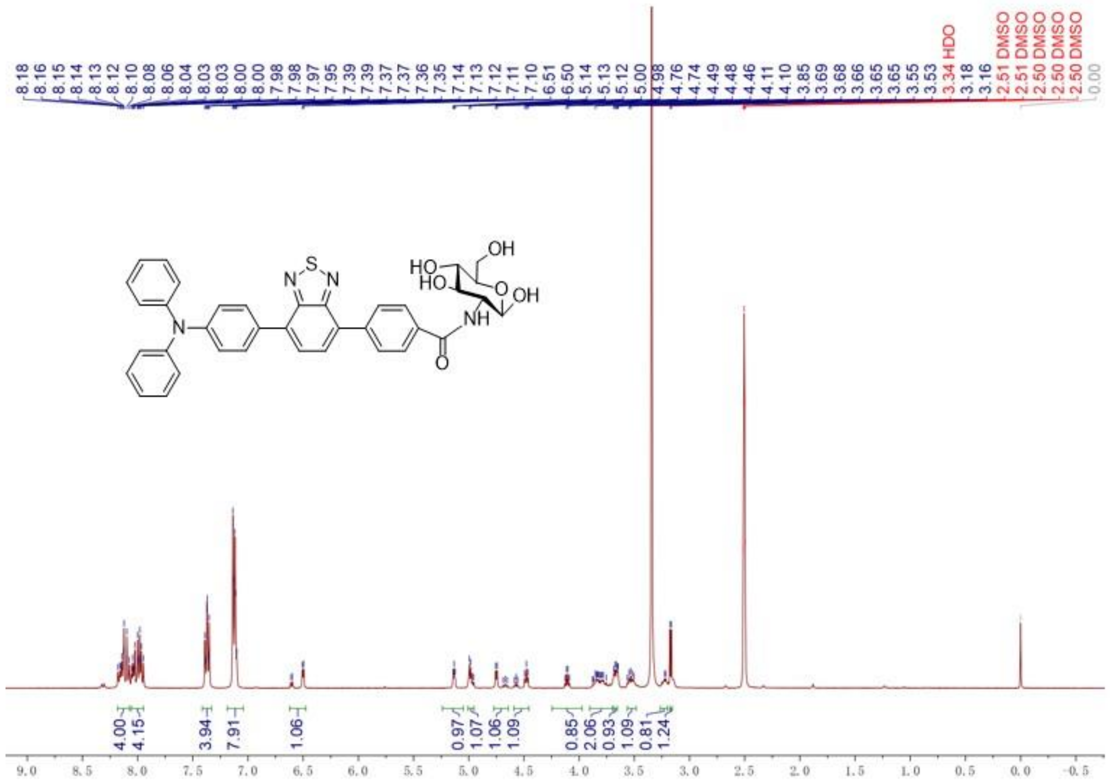

Figure S9. ^{1}H NMR spectrum of TB-GlcN in DMSO-d_6.

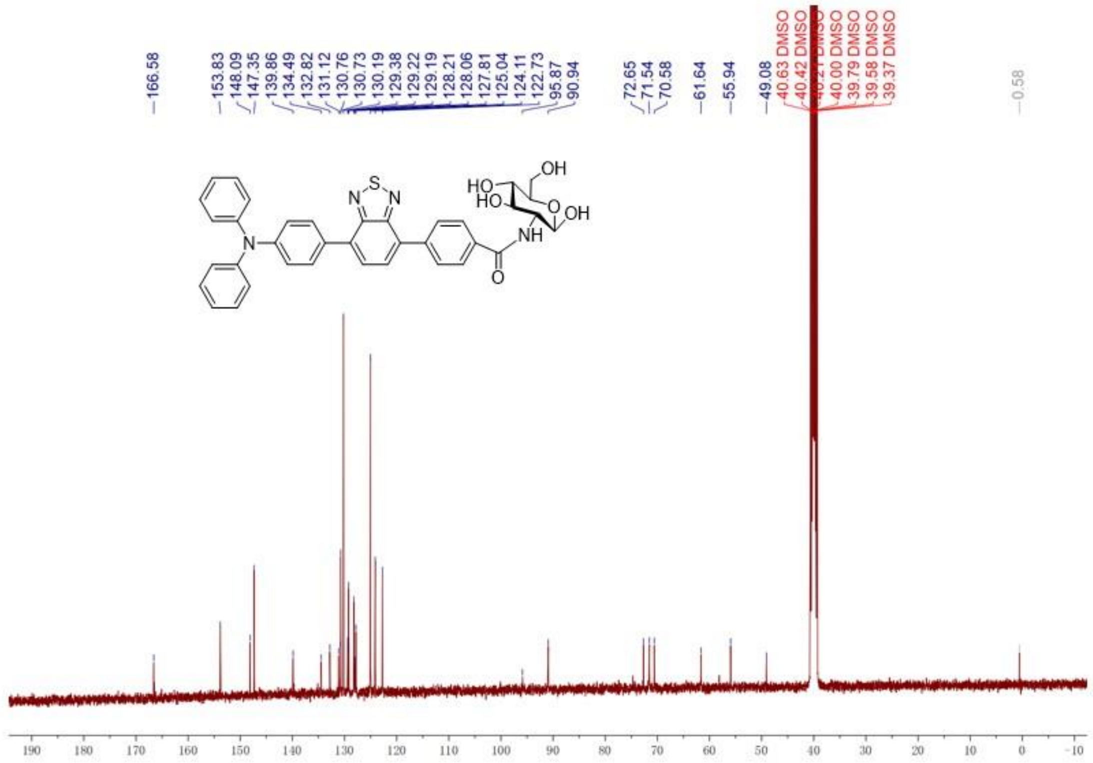

Figure S10. ^{13}C NMR spectrum of TB-GlcN in DMSO-d_6.

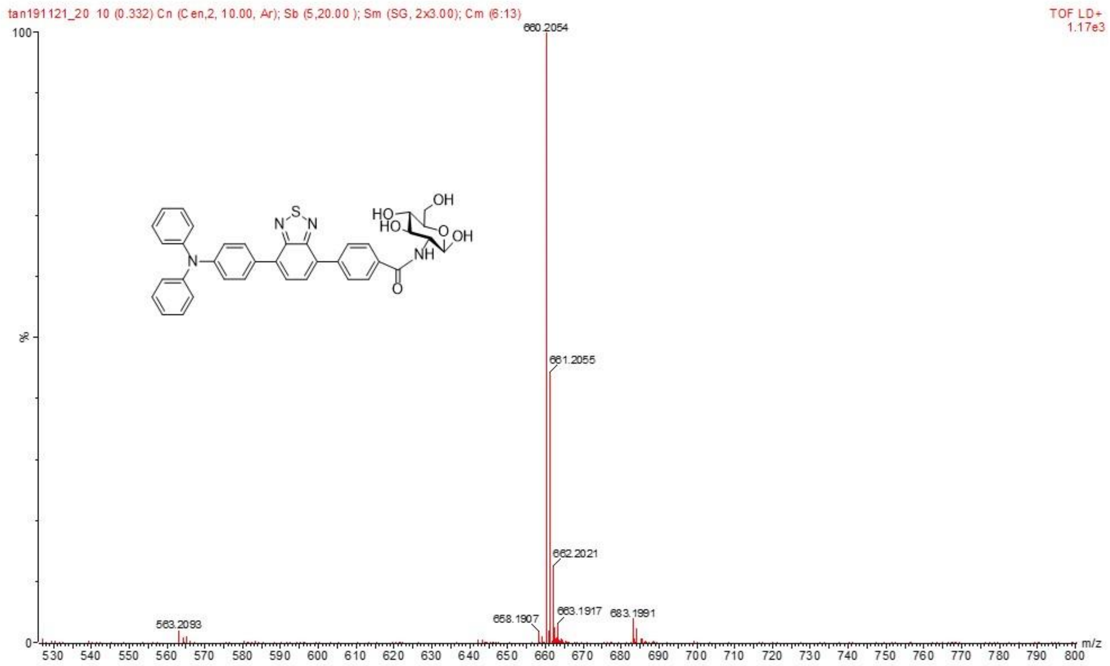

Figure S11. HRMS of compound TB-GlcN.

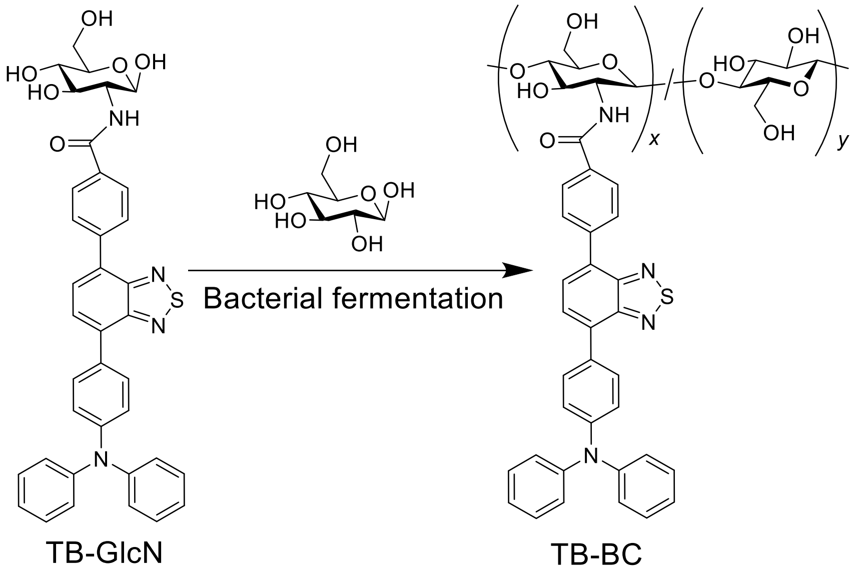

Scheme S2. Biological synthesis of TB–BC through bacterial fermentation.

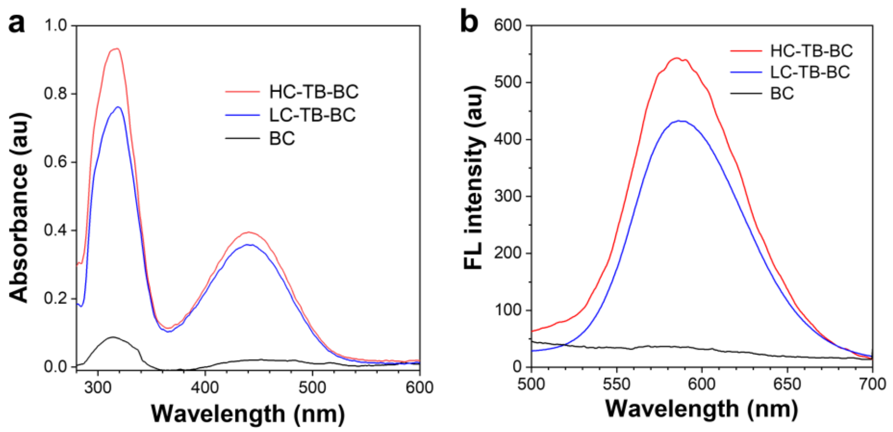

Figure S12. (a) Absorption and (b) PL spectra of HC-TB–BC, LC-TB–BC and BC.

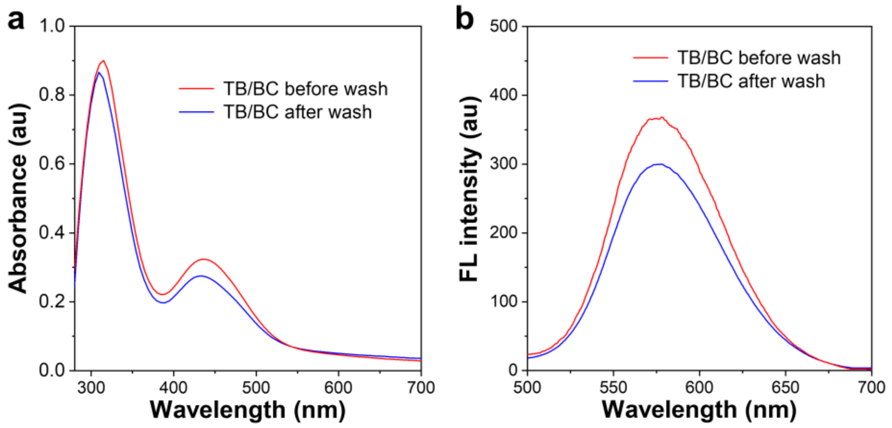

Figure S13. (a) Absorption and (d) PL spectra of TB/BC before and after washing with NaOH (2%, w/v).

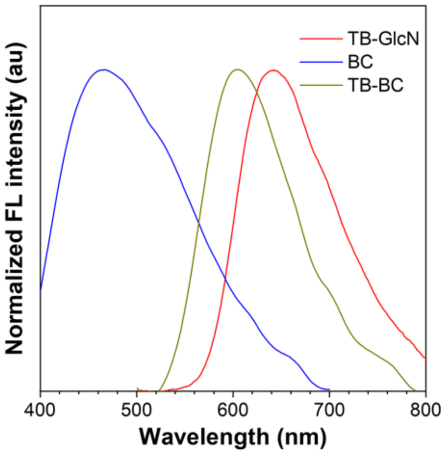

Figure S14. Normalized PL spectra of TB-GlcN powder, BC film and TB–BC film.

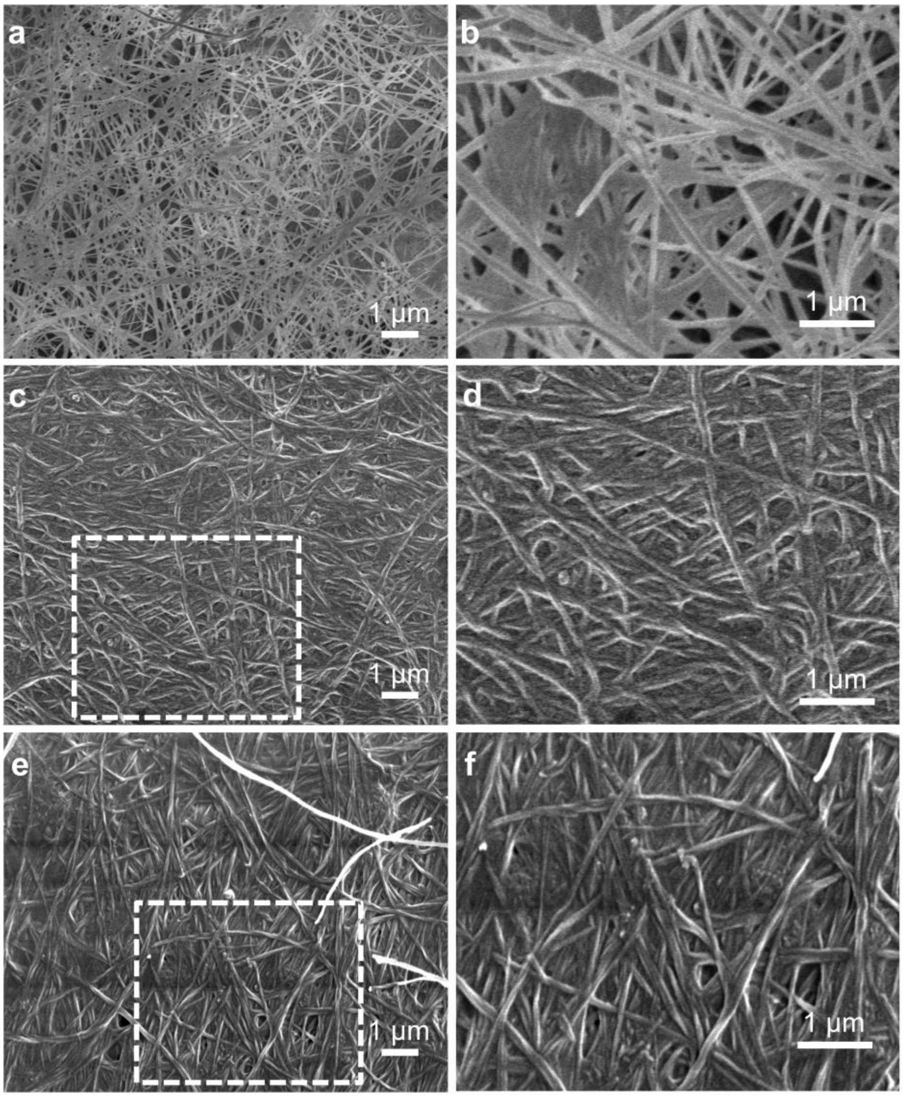

Figure S15. SEM photographs of (a, b) BC, (c, d) HC-TB–BC and (e, f) LC-TB–BC.

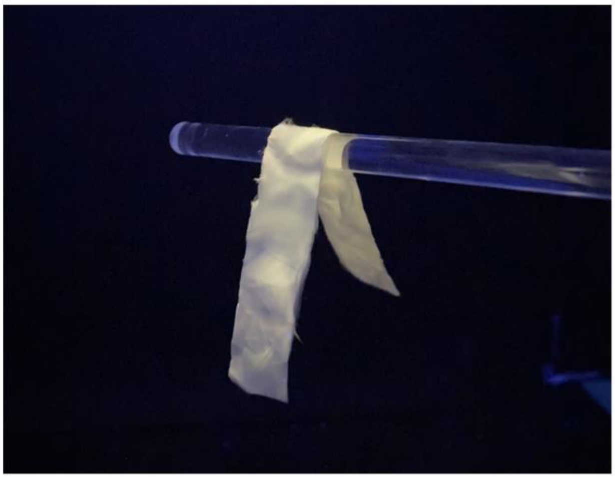

Figure S16. The fluorescence photo of TB–BC/PVP mat through electrospinning.

Reference

(1) Chen, X.; Yang, Z.; Li, W.; Mao, Z.; Zhao, J.; Zhang, Y.; Wu, Y.; Jiao, S.; Liu, Y.; Chi, Z. Nondoped Red Fluorophores with Hybridized Local and Charge-Transfer State for High-Performance Fluorescent White Organic Light-Emitting Diodes. *ACS Appl. Mater. Interfaces* **2019**, *11*, 39026-39034.
